# Association of mycophenolic acid dose with efficacy and safety events in kidney transplant patients receiving tacrolimus: an analysis of the Mycophenolic acid Observational REnal transplant registry

**DOI:** 10.1111/ctr.12035

**Published:** 2012-11-02

**Authors:** Cataldo Doria, Stuart Greenstein, Mohanram Narayanan, Kimi Ueda, Anne Wiland, Kevin McCague, Bashir Sankari, Laurence Chan

**Affiliations:** a Department of Transplant Surgery, Thomas Jefferson University HospitalPhiladelphia, PA, USA; b Department of Surgery, Montefiore Medical CenterNew York, NY, USA; c Department of Medicine, Scott and White Memorial HospitalTemple, TX, USA; d The Barry S. Levin, M.D. Department of Transplantation, California Pacific Medical CenterSan Francisco, CA, USA; e Novartis Pharmaceuticals CorporationEast Hanover, NJ, USA; f Department of Urology, Charleston Area Medical CenterCharleston, WV, USA; g Department of Medicine, University of Colorado Medical CenterDenver, CO, USA

**Keywords:** immunosuppression, kidney transplantation, mycophenolic acid, outcomes

## Abstract

*Background* Dose-finding studies for mycophenolic acid (MPA) in tacrolimus-treated kidney transplant patients are lacking.

*Methods* Data from 901 *de novo* kidney transplant recipients enrolled in the prospective, non-interventional Mycophenolic acid Observational REnal (MORE) transplant registry were analyzed according to baseline daily MPA dose (<2000, 2000 or >2000 mg).

*Results* The proportion of patients receiving 2000 and <2000 mg was 77.6% and 19.9% at baseline, 74.5% and 23.3% at month 1, 62.4% and 35.5% at month 3, 48.5% and 50.2% at month 6, and 44.1% and 55.2% at month 12. More patients were maintained on 2000 mg with enteric-coated mycophenolate sodium (EC-MPS) vs. mycophenolate mofetil (month 6, 52.7% vs. 43.0% [p = 0.02]; month 12, 47.3% vs. 39.4% [p = 0.08]). Multivariate modeling showed no significant effect of baseline MPA dose on 12-month risk of biopsy-proven acute rejection, graft loss or estimated GFR, or on safety events including MPA discontinuation other than a higher rate of gastrointestinal adverse events in patients with an initial MPA dose >2000 mg (p = 0.029) vs. 2000 mg.

*Conclusions* These findings suggest that an initial MPA dose of <2000 mg does not compromise 12-month efficacy in tacrolimus-treated kidney transplants, but controlled trials are required and the lower threshold for MPA dose remains to be defined.

Mycophenolic acid (MPA) has become a well-established component of immunosuppression regimens following pivotal trials with the mycophenolate mofetil (MMF) formulation in the mid-1990s 1–3. These studies, all conducted in patients receiving concomitant cyclosporine, led to the recommendation that MMF be administered at a fixed daily dose of 2000 mg 4. Since then, cyclosporine has largely been replaced by tacrolimus as *de novo* immunosuppression after kidney transplantation 5. As cyclosporine and tacrolimus influence the enterohepatic circulation and metabolism of MPA differently 6, MPA exposure is higher in patients receiving concomitant tacrolimus compared with cyclosporine 7–9, with the estimates of difference ranging from 19% 7 to 46% 8. Dose-finding studies for MPA in combination with tacrolimus are, unfortunately, lacking although the CLEAR study investigated use of an early loading dose of MMF in tacrolimus-treated patients 10. Some centers empirically initiate MPA at a dose below 2000 mg in kidney transplant patients receiving tacrolimus, but although retrospective clinical 11–13 and registry 14–17 analyses have shown an increased risk of acute rejection 11,12 and graft loss 13–17 following MPA dose reductions 13–16 or discontinuation 14–17, the effect of a lower MPA dose from time of transplant remains largely unexamined. Moreover, these retrospective analyses have generally not been restricted to tacrolimus-treated patients. Also, there are limited data to suggest that the enteric-coated mycophenolate sodium (EC-MPS) formulation of MPA, in which MPA release is delayed with the aim of improving gastrointestinal tolerability, may permit maintenance of higher MPA dosing than MMF in tacrolimus-treated patients 18, but this has not been investigated from the time of transplantation.

Data collected prospectively within the Mycophenolic acid Observational REnal (MORE) transplant registry were used to evaluate efficacy and safety events during the first year after kidney transplantation in patients receiving MPA and tacrolimus. Results were analyzed according to the initial MPA dose and MPA dose over time and stratified according to use of MMF or EC-MPS.

## Methods

### Study design

The MORE Registry is a multicenter, prospective, observational study of *de novo* renal transplant patients receiving MPA therapy (either EC-MPS or MMF) as part of their immunosuppressive regimen at 40 transplant centers in the US. Eligible sites were selected to meet geographical and size diversity. The study is performed under routine clinical conditions according to local practice. Recruitment started in June 2007 and closed in May 2010. Data collection is ongoing. The MORE Registry is conducted according to the Declaration of Helsinki, with informed consent obtained for all enrolled patients.

Standard data obtained routinely during clinic visits are recorded prospectively from baseline (defined as within two wk of transplantation) at months 1, 3, 6, and 12 and annually thereafter for the first four yr post-transplant. Information is entered by designated investigator staff to a Web-based electronic data capture system with real-time data validation checks to ensure data quality and are entered directly into the study database. Data undergo an automated data quality review followed by data management review and both electronic and on-site monitoring.

### Patients and immunosuppression

To minimize the possibility of center-imposed bias, investigators sought participation of all *de novo* renal transplant recipients seen at the study site within two wk of transplantation who met eligibility criteria. Patients ≥18 yr who were receiving MPA at time of discharge from hospital following kidney transplantation from a deceased or living donor were eligible for enrollment. Non-inclusion criteria were (i) multiorgan transplantation or a current or planned non-kidney graft, (ii) enrolled or planning to enroll in an investigational clinical trial involving an immunosuppressive agent that was either blinded or unapproved by the Food and Drug Administration, and (iii) unlikely to have up to five yr follow-up data available for the study. Participating centers were selected for geographic and size diversity, with investigators seeking participation of all eligible *de novo* kidney transplant recipients at the center. The current analysis was restricted to patients receiving tacrolimus at baseline.

### MPA administration

The choice of MPA treatment (EC-MPS or MMF) is determined by the center and/or physician-specific protocols. Enrollment targets were capped at approximately 2:1 (EC-MPS:MMF), with each center permitted to enroll a maximum of 36 EC-MPS treated patients and 18 MMF-treated patients. Doses of EC-MPS or MMF are initiated and adjusted according to local practice.

For the purposes of analysis, MPA daily doses were classified as <2000, 2000, and >2000 mg. EC-MPS dose was recalculated to the MMF equivalent by multiplying by 1.3889 19. The reason for MPA intolerance, defined as dose reduction, interruption, or discontinuation, was recorded.

### Statistical methods

Data on MPA dosing, including maintenance of full recommended dose, mean dose, and changes from baseline; incidence and reasons for MPA intolerance; and incidence of selected adverse events, are presented descriptively according to baseline MPA dose (<2000, 2000 and >2000 mg) and for EC-MPS vs. MMF administration at baseline. Fisher's exact test was used to compare the proportion of patients maintained on the full recommended MPA dose between the EC-MPS and MMF groups. Student's *t*-test was used to compare mean doses between EC-MPS and MMF. First-order Markov transition probabilities were estimated to illustrate patient movement from one MPA dose group to another from the start to the end of specified periods (baseline to month 1, months 1–3, months 3–6, and months 6–12). A Cox proportional hazards model 1972 was used to analyze time to biopsy-proven acute rejection (BPAR), graft failure, and MPA discontinuation within the first year post-transplant according to baseline MPA dose. A complementary log–log regression model 21 was used to analyze time to first cytomegalovirus (CMV) infection as an adverse event, BK virus (BKV) infection as an adverse event, gastrointestinal adverse events, leukopenia (white blood cell count <4000/mm^3^) and thrombocytopenia (platelets <50 000/mm^3^) according to baseline MPA dose, to accommodate interval censoring because the exact date of occurrence of these events was not recorded. A linear mixed-effects random intercept model 22, with patient included as a random effect, was used to analyze estimated GFR [eGFR, MDRD formula 23] and serum creatinine over time. All models included recipient age (per one yr increase), race (African American, white, other), gender, primary reason for transplantation (diabetes mellitus, hypertension or other), donor age (per one yr increase), delayed graft function, type of donor (deceased vs. living donor), and baseline concomitant immunosuppression (calcineurin inhibitor [tacrolimus vs. cyclosporine], corticosteroids, antibody induction). Missing data were not imputed in any analysis.

Analyses were performed using SAS statistical software (SAS Institute, Cary, NC, USA). p Values <0.05 were considered statistically significant.

## Results

### Patient population and baseline immunosuppression

In total, 901 tacrolimus-treated patients from 40 participating centers were included in the analysis. Seventeen patients within the data set who were receiving cyclosporine were excluded. EC-MPS was administered at baseline in 613 patients (68.0%), with MMF in the remaining 288 patients (32.0%). The majority of patients (77.6%, n = 699) received a baseline MPA dose of 2000 mg, with a further 19.9% (n = 179) receiving <2000 mg. Recipient and donor characteristics showed no marked differences between patients receiving an MPA dose of 2000 or <2000 mg at baseline (Table 1). An initial dose >2000 mg was preferentially used in African American patients and retransplants, a finding that would be expected in view of the higher immunological risk of these subpopulations. Among patients with a baseline MPA dose <2000 mg, baseline mean tacrolimus trough concentration was lower and maintenance corticosteroids were used less frequently than in the group receiving MPA 2000 mg at baseline. Median steroid dose was similar between groups (p = 0.39) (Table 1). During the study, 85 patients discontinued tacrolimus therapy, 21 of whom were switched to cyclosporine, and 55 of whom started sirolimus therapy.

**Table 1:** Baseline characteristics and concomitant immunosuppression according to baseline MPA dose

	Baseline MPA dose^a^				
<2000 mg	2000 mg	>2000 mg	All	
N = 179	N = 699	N = 23	N = 901	p Value
*Recipient*					
Age (yr), mean (SD)	53 (13)	51 (14)	46 (13)	52 (14)	0.05
Male	111 (62.0%)	448 (64.1%)	17 (73.9%)	576 (63.9%)	0.53
African American	47 (26.3%)	155 (22.2%)	15 (65.2%)	217 (24.1%)	<0.01
Previous kidney transplant	14 (7.8%)	63 (9.0%)	4 (17.4%)	81 (9.0%)	0.32
Reason for transplantation					0.81
Hypertension/nephrosclerosis	44 (24.6%)	154 (22.0%)	7 (30.4%)	205 (22.8%)	
Diabetes mellitus	36 (20.1%)	170 (24.3%)	2 (8.7%)	208 (23.1%)	
Polycystic disease	22 (12.3%)	78 (11.2%)	2 (8.7%)	102 (11.3%)	
Glomerulonephritis/glomerular disease	23 (12.9%)	106 (15.2%)	4 (17.4%)	133 (14.8%)	
Other	30 (16.8%)	78 (11.2%)	4 (17.4%)	112 (12.4%)	
Peak panel reactive antibody <30%	113/133 (85.0%)	533/651 (81.9%)	16/20 (80.0%)	662/804 (82.3%)	0.67
Delayed graft function	27/160 (16.9%)	108/694 (15.6%)	1/23 (4.4%)	136/877 (15.5%)	0.30
*Donor*					
Age (yr), mean (SD)	43 (15)	41 (15)	41 (14)	41 (15)	0.15
Age ≥60 yr	23 (12.9%)	63 (9.0%)	2 (8.7%)	88 (10.0%)	0.31
Type of donor					0.23
Brain death	64 (35.8%)	321 (45.9%)	10 (43.5%)	395 (43.8%)	
Donation after cardiac death	32 (17.9%)	88 (12.6%)	2 (8.7%)	122 (13.5%)	
Living related	45 (24.1%)	157 (22.5%)	7 (30.4%)	209 (23.2%)	
Living unrelated	38 (21.2%)	133 (19.0%)	4 (17.4%)	175 (19.4%)	
Expanded criteria	29 (16.3%)	77 (11.0%)	2 (8.7%)	108 (12.0%)	0.14
Cold ischemia time (h), mean (SD)	10.8 (10.2)	10.4 (9.8)	13.4 (12.8)	10.5 (10.0)	0.46
*Induction therapy*					
Basiliximab	40 (22.4%)	137 (19.6%)	1 (4.4%)	178 (19.8%)	0.12
Daclizumab	1 (0.6%)	51 (7.3%)	0	52 (5.8%)	<0.01
Rabbit antithymocyte globulin	78 (43.6%)	447 (64.0%)	16 (69.6%)	541 (60.0%)	<0.01
Alemtuzumab	54 (30.2%)	58 (8.3%)	2 (8.7%)	114 (12.7%)	<0.01
*Maintenance immunosuppression*					
MPA					
EC-MPS	112 (62.6%)	488 (69.8%)	13 (56.5%)	613 (68.0%)^a^	0.25
MMF	67 (37.4%)	211 (30.2%)	10 (43.5%)	288 (32.0%)^b^	
MPA dose (mg/d), mean (SD)					
Month 0	1179 (341)	2000 (0)	2917 (222)	1860 (400)	<0.01
Month 1	1403 (435)	1922 (294)	2283 (580)	1832 (400)	<0.01
Month 3	1360 (497)	1801 (413)	1964 (759)	1719 (476)	<0.01
Month 6	1302 (486)	1624 (501)	1513 (868)	1561 (525)	<0.01
Month 12	1323 (509)	1548 (501)	1423 (760)	1507 (516)	<0.01
Tacrolimus trough level (ng/mL), mean (SD)^a^					
Month 0	7.1 (4.6)	9.1 (4.9)	8.1 (5.6)	8.7 (4.9)	<0.01
Month 1	8.8 (3.7)	9.7 (4.5)	9.9 (3.3)	9.5 (4.5)	0.05
Month 3	8.5 (3.6)	9.4 (3.9)	10.3 (3.0)	9.2 (3.8)	0.02
Month 6	7.9 (3.6)	8.0 (3.1)	8.1 (3.2)	8.0 (3.2)	0.98
Month 12	7.1 (3.2)	7.6 (2.9)	7.6 (2.3)	7.6 (3.0)	0.29
Corticosteroids					
Yes	71 (40.0%)	433 (62.0%)	6 (26.1%)	510 (56.6%)	<0.01
Dose (mg/d), median (range)	20 (5–620)	25 (2–500)	20 (10–30)	25 (2–620)	0.39

MPA, mycophenolic acid; EC-MPS, enteric-coated mycophenolate sodium.

EC-MPS dose was recalculated to the MMF equivalent by multiplying by 1.3889 19.

a Thirty-eight patients switched from MMF to EC-MPS; 36 patients switched from MMF (Cellcept®) to generic MMF; two patients switched from MMF (Cellcept®) to EC-MPS then to generic MMF.

b Twelve patients switched from EC-MPS to MMF (Cellcept®); five patients switched to generic MMF; one patient switched from EC-MPS to MMF (Cellcept®) then to generic MMF.

Use of rabbit antithymocyte globulin (rATG) was lower, and administration of alemtuzumab was higher, in the cohort receiving <2000 mg at baseline vs. those given 2000 mg (Table 1). The mean (SD) total dose of rATG was similar in the two groups (373 [151] mg in the cohort receiving MPA <2000 mg and 386 [155] mg among patients receiving MPA 2000 mg), but higher in the group with a baseline MPA dose >2000 mg (518 [143] mg).

The mean (SD) starting dose of MPA was 1866 (389) mg with EC-MPS, and 1847 (425) mg with MMF. Baseline characteristics were similar in the EC-MPS and MMF groups, with no marked differences in the use of lymphocyte-depleting induction (EC-MPS: rATG 62.2%, alemtuzumab 11.6%; MMF: rATG 55.6%, alemtuzumab 14.9%).

### MPA dose changes

The proportion of patients receiving MPA 2000 mg decreased steadily over the first year post-transplant (74.5%, 62.4%, 48.5%, and 44.1% at months 1, 3, 6, and 12, respectively), with an associated increase in the proportion receiving <2000 mg (23.3%, 35.5%, 50.2%, and 55.2%, respectively). Among patients receiving MPA 2000 mg at baseline, between 10% and 24% switched to a lower dose during months 1, 1–3, 3–6, and 6–12 with no clear change in propensity to switch to a lower dose over time (Table 2). The mean dose among patients initially given a dose below 2000 mg was in the range 1172–1387 mg throughout the first year. In contrast, almost a quarter of patients initially receiving <2000 mg at baseline shifted to a higher dose during the first month post-transplant (39/165, 23.6%) (Table 2).

**Table 2:** Transitions between MPA dose categories during specific time intervals (first-order Markov transition probabilities)

MPA dose at start of period	MPA dose at end of period		
	<2000 mg	2000 mg	>2000 mg
*Transitions during baseline to month 1*			
<2000 mg (n = 165)	126 (76.4%)	39 (23.6%)	0
2000 mg (n = 675)	71 (10.5%)	594 (88.0%)	10 (1.5%)
>2000 mg (n = 23)	4 (17.4%)	10 (43.5%)	9 (39.1%)
*Transitions from month 1 to month 3*			
<2000 mg (n = 184)	170 (92.4%)	12 (6.5%)	2 (1.1%)
2000 mg (n = 608)	113 (18.6%)	489 (80.4%)	6 (1.0%)
>2000 mg (n = 19)	3 (15.8%)	7 (36.8%)	9 (47.4%)
*Transitions from month 3 to month 6*			
<2000 mg (n = 247)	241 (97.6%)	6 (2.4%)	0
2000 mg (n = 450)	109 (24.2%)	339 (75.3%)	2 (0.4%)
>2000 mg (n = 13)	2 (15.4%)	4 (30.8%)	7 (53.9%)
*Transitions from month 6 to month 12*			
<2000 mg (n = 282)	260 (92.2%)	22 (7.8%)	0
2000 mg (n = 285)	56 (19.7%)	229 (80.4%)	0
>2000 mg (n = 8)	0	4 (50.0%)	4 (50.0%)

MPA, mycophenolic acid; EC-MPS, enteric-coated mycophenolate sodium.

EC-MPS dose was recalculated to the MMF equivalent by multiplying by 1.3889 19.

The most frequent reasons for MPA intolerance (i.e., dose reduction, interruption or discontinuation) were hematologic adverse events, gastrointestinal symptoms, and viral infections (Table 3). There were no consistent differences in the reasons for MPA intolerance between patients with a baseline dose of <2000 mg vs. 2000 mg.

**Table 3:** Reasons for MPA intolerance (defined as dose reduction, interruption, or discontinuation)

MPA dose at baseline	<2000 mg				2000 mg				>2000 mg			
	BL – month 1 (n = 168)	Months 1–3 (n = 165)	Months 3–6 (n = 147)	Months 6–12 (n = 116)	BL – month 1 (n = 686)	Months 1–3 (n = 673)	Months 3–6 (n = 634)	Months 6–12 (n = 555)	BL – month 1 (n = 23)	Months 1–3 (n = 22)	Months 3–6 (n = 20)	Months 6–12 (n = 16)													
Hematologic	7 (4.2)	16 (9.7)	13 (8.8)	2 (1.7)	18 (2.6)	58 (8.6)	78 (12.3)	29 (5.2)	3 (13.0)	4 (18.2)	2 (10.0)	1 (6.3)
Gastrointestinal	6 (3.6)	7 (4.2)	4 (2.7)	2 (1.7)	37 (5.4)	41 (6.1)	27 (4.3)	17 (3.1)	4 (17.4)	1 (4.5)	2 (10.0)	0 (0.0)
Viral infection	3 (1.8)	4 (2.4)	5 (3.4)	4 (3.4)	2 (0.3)	17 (2.5)	40 (6.3)	31 (5.6)	1 (4.3)	1 (4.5)	1 (5.0)	0 (0.0)
CMV	0 (0.0)	1 (0.6)	2 (1.4)	2 (1.7)	1 (0.1)	4 (0.6)	12 (1.9)	12 (2.2)	0 (0.0)	0 (0.0)	0 (0.0)	0 (0.0)
BK virus	3 (1.8)	4 (2.4)	3 (2.0)	2 (1.7)	1 (0.1)	13 (1.9)	30 (4.7)	20 (3.6)	1 (4.3)	1 (4.5)	1 (5.0)	0 (0.0)

BL, baseline; MPA, mycophenolic acid; CMV, cytomegalovirus.

Values are shown as n (%).

**Figure 1:**
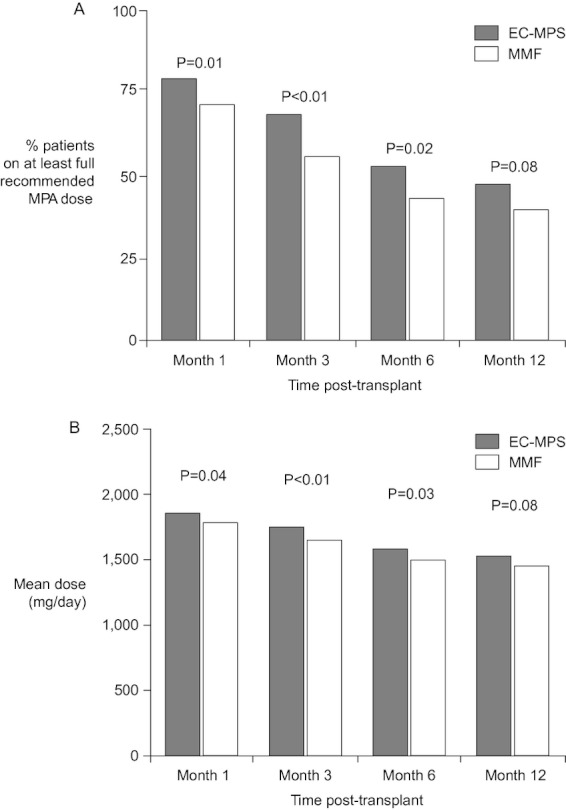
(A) Proportion of patients maintained on at least the full recommended dose of EC-MPS (≥1440 mg/d) or MMF (≥2000 mg/d) (Fisher's exact test) and (B) mean dose of EC-MPS and MMF (Student's *t*-test). EC-MPS dose was recalculated to the MMF equivalent by multiplying by 1.3889 19. Vertical bars indicate SD values.

A higher proportion of patients receiving EC-MPS were maintained on at least the full recommended dose compared to those receiving MMF, a difference that was significant to month 6 post-transplant (Fig 1A). Correspondingly, patients in the EC-MPS group received significantly higher mean MPA doses than those given MMF (Fig 1B). There were no marked differences in the reasons for MPA intolerance between patients receiving EC-MPS vs. MMF (Table 4).

**Table 4:** Reasons for MPA intolerance (defined as dose reduction, interruption, or discontinuation) according to MPA formulation

	EC-MPS				MMF			
	BL – month 1 (n = 595)	Months 1–3 (n = 580)	Months 3–6 (n = 550)	Months 6–12 (n = 466)	BL – month 1 (n = 282)	Months 1–3 (n = 280)	Months 3–6 (n = 251)	Months 6–12 (n = 221)
Hematologic	20 (3.4)	49 (8.4)	62 (11.3)	19 (4.1)	8 (2.8)	29 (10.4)	31 (12.4)	13 (5.9)
Gastrointestinal	36 (6.1)	31 (5.3)	27 (4.9)	15 (3.2)	11 (3.9)	18 (6.4)	6 (2.4)	4 (1.8)
Viral infection	5 (0.8)	16 (2.8)	31 (5.6)	23 (4.9)	1 (0.4)	6 (2.1)	15 (6.0)	12 (5.4)
CMV	1 (0.2)	4 (0.7)	10 (1.8)	10 (2.1)	0 (0.0)	1 (0.4)	4 (1.6)	4 (1.8)
BK virus	4 (0.7)	13 (2.2)	22 (4.0)	13 (2.8)	1 (0.4)	5 (1.8)	12 (4.8)	9 (4.1)

BL, baseline; MPA, mycophenolic acid; EC-MPS, enteric-coated mycophenolate sodium; MMF, mycophenolate mofetil.

EC-MPS dose was recalculated to the MMF equivalent by multiplying by 1.3889 19. Values are shown as n (%).

### Efficacy and renal function

The overall incidence of BPAR at month 12 was 9.1% (9.5% for patients receiving EC-MPS, 8.0% with MMF [p = 0.298]). When analyzed according to baseline MPA dose, the 12-month incidence of BPAR was 7.0% with MPA <2000 mg, 9.2% for MPA 2000 mg, and 18.5% for MPA >2000 mg. Cox regression modeling showed no significant effect of baseline MPA dose on the 12-month risk of BPAR when adjusted for recipient and donor factors (Table 5). Graft loss at month 12 occurred in 2.4% of patients with baseline MPA dose <2000 mg, 2.6% with MPA 2000 mg, and 11.5% with MPA >2000 mg. There was no significant association between baseline MPA dose and graft loss on multivariate analysis (Table 5).

**Table 5:** Multivariate analyses (Cox regression modeling) of association between MPA dose and efficacy and safety events at month 12

	MPA dose (mg)	HR	95% CI	p Value
*Efficacy events*				
BPAR^a^	<2000	0.70	0.36–1.38	0.303
>2000	1.52	0.60–3.89	0.379
Graft loss^a^	<2000	1.11	0.55–2.26	0.771
>2000	1.29	0.30–5.54	0.731
*Safety events*				
CMV infection^b^	<2000	0.63	0.28–1.39	0.253
>2000	–c		–
BKV infection^b^	<2000	0.64	0.35–1.15	0.133
>2000	0.67	0.16–2.77	0.578
Leukopenia^b^	<2000	1.03	0.77–1.37	0.852
>2000	1.74	0.93–3.25	0.082
Gastrointestinal adverse events^b^	<2000	0.92	0.74–1.16	0.490
>2000	1.77	1.06–2.96	0.029
MPA discontinuation^a^	<2000	0.77	0.44–1.33	0.344
>2000	0.75	0.18–3.10	0.686

MPA, mycophenolic acid; HR, hazard ratio; CI, confidence interval; BPAR, biopsy-proven acute rejection; eGFR, estimated GFR (MDRD formula).

EC-MPS dose was recalculated to the MMF equivalent by multiplying by 1.3889 19. Patients receiving 2000 mg MPA were the reference group (i.e., 1.00).

a Cox proportional hazard model.

b Complementary log-log regression analysis.

c No cases of CMV infection.

Mean (SD) eGFR at 12 months post-transplant was 57.9 (19.7) mL/min/1.73 m^2^ for patients with baseline MPA dose <2000 mg (n = 111), 59.0 (19.8) mL/min/1.73 m^2^ for MPA 2000 mg (n = 550), and 48.5 (18.6) mL/min/1.73 m^2^ for MPA >2000 mg at baseline (n = 16). The linear mixed-effects model showed no evidence of a significant association between baseline MPA dose and eGFR or serum creatinine over time (<2000 mg vs. 2000 mg: p = 0.546 for eGFR and p = 0.547 for serum creatinine; >2000 mg vs. 2000 mg: p = 0.510 and 0.691, respectively).

### Safety

There was no evidence of a significant effect of baseline MPA dose on risk of CMV or BKV infection, although there was a non-significant trend (p = 0.082) to higher risk of leukopenia with an MPA dose >2000 mg (Table 5). This may have been influenced by the higher rate of rATG induction in patients receiving MPA >2000 mg (Table 1). Use of other therapies that may induce leukopenia (acyclovir, ganciclovir, valganciclovir, and intravenous immunoglobulin) was similar between dosage groups other than valganciclovir, which was administered in 72.1%, 71.8%, and 95.7% of patients receiving an initial MPA dose <2000, 2000, or >2000 mg, respectively. Only six cases of thrombocytopenia were reported, so no analysis of thrombocytopenia was performed. There was a higher rate of gastrointestinal adverse events in the cohort with an initial MPA dose >2000 mg (p = 0.029) vs. those given a dose of 2000 mg, although the number of patients with baseline dose >2000 mg was small. Multivariate analysis showed that MPA dose at baseline was not associated with risk of MPA discontinuation by one yr across all patients (Table 5) or within the EC-MPS or MMF subpopulations (data not shown). There was no consistent pattern of difference in any safety event between the EC-MPS and MMF cohorts at months 1, 3, 6, and 12 (Table 6). Discontinuation of study drug occurred in 11.1% (68/613) patients receiving EC-MPS and 10.1% (29/288) of MMF-treated patients by month 12.

**Table 6:** Incidence of MPA discontinuation and selected adverse events in patients receiving EC-MPS or MMF

	Month 1		Month 3		Month 6		Month 12	
	EC-MPS	MMF	EC-MPS	MMF	EC-MPS	MMF	EC-MPS	MMF
MPA discontinuation	6/595 (1.0)	2/282 (0.7)	21/578 (3.6)	11/277 (4.0)	35/542 (6.5)	23/244 (9.4)	51/460 (11.1)	22/218 (10.1)
Cytomegalovirus infection	1/595 (0.2)	0/282 (0.0)	11/580 (1.9)	3/280 (1.1)	20/550 (3.6)	8/251 (3.2)	21/466 (4.5)	8/222 (3.6)
BK virus infection	5/595 (0.8)	2/282 (0.7)	19/580 (3.3)	11/280 (3.9)	39/550 (7.1)	16/251 (6.4)	30/466 (6.4)	17/222 (7.7)
Gastrointestinal adverse events	231/595 (38.8)	126/282 (44.7)	188/580 (32.4)	98/280 (35.0)	171/550 (31.1)	69/251 (27.5)	149/466 (32.0)	60/222 (27.0)
Leukopenia	75/595 (12.6)	42/282 (14.9)	132/580 (22.8)	73/280 (26.1)	139/550 (25.3)	66/251 (26.3)	65/466 (14.0)	37/222 (16.7)

MPA, mycophenolic acid; EC-MPS, enteric-coated mycophenolate sodium; MMF, mycophenolate mofetil.

Values are shown as n/N (%).

## Discussion

In this prospective analysis, there was no association between initial MPA dose and the one-yr incidence of BPAR or other efficacy endpoints in kidney transplant patients receiving tacrolimus-based immunosuppression, based on a threshold of 2000 mg/d. It should be borne in mind, however, that the number of patients in the highest dosing group (initial dose >2000 mg) was small. In this real-life population, results also highlighted the proportion of tacrolimus-treated patients in whom the initial recommended dose of 2000 mg could be tolerated, with the dose being reduced in approximately 40% of cases during the first year post-transplant.

The absence of an effect of initial MPA dose on risk of rejection does not appear to be due to selective use of lower doses in patients at low immunological risk. The proportion of African Americans and patients with PRA <30% was similar in the cohorts receiving <2000 mg or 2000 mg MPA at time of transplant, as was the proportion of living donors and the duration of cold ischemia (Table 1). It may be relevant that the analysis used an initial MPA dose of 2000 mg as the threshold for analysis. In a retrospective study of 547 kidney transplant patients in the UK, Shah et al. 2008 found the rate of graft survival to decrease significantly only when the MPA dose fell to below 1000 mg, with no difference between 1000 and 2000 mg compared to ≥2000 mg (approximately half the patients were receiving cyclosporine, and half tacrolimus).

It is also likely that concomitant use of tacrolimus may have contributed to the lack of association between baseline MPA dose and efficacy outcomes. In an analysis of Collaborative Transplant data on 8439 low-risk kidney transplant patients receiving tacrolimus and MMF during the second year post-transplant, Opelz et al. 2008 showed a significant association between graft survival and MMF dose ≤1000 mg vs. 1001–2000 mg in patients receiving cyclosporine, but no significant association with MMF in patients receiving tacrolimus even using a lower cutoff point of MPA dose ≤500 mg vs. 501-2000 mg. For a given dose, MPA plasma concentration is lowered in the presence of cyclosporine due to decreased biliary secretion of the primary metabolite MPA glucuronide 6. In patients given tacrolimus, MPA exposure is increased relative to cyclosporine-treated patients 7–9, partly due to the lack of inhibition of enterohepatic circulation of MPA but possibly also as a consequence of a drug–drug interaction with MPA metabolism 24. Clinically, this can permit lower MPA dosing in tacrolimus-treated kidney transplant patients without loss of efficacy. In the recent DIRECT study, 682 *de novo* kidney transplant recipients were randomized to cyclosporine or tacrolimus with MPA 25. Despite a median MPA dose of 1389 mg (MMF equivalents) in the tacrolimus group compared to 2000 mg in the cyclosporine group, the rate of BPAR at month 6 was similar in both arms.

Analysis of the association between MPA dose and adverse events revealed a significantly higher rate of gastrointestinal events in patients receiving an initial MPA dose >2000 mg. Dose dependency of MPA-related gastrointestinal adverse events has been reported elsewhere 26, possibly as a result of local gut toxicity 27. There was also a non-significant trend to a higher frequency of leukopenia in the highest initial MPA dose group, even after adjustment for use of lymphocyte-depleting induction, but this finding should be interpreted cautiously due to the low number of patients.

The finding that mean MPA dose was higher in patients receiving EC-MPS vs. MMF is consistent with previous retrospective 2 and prospective 18–29 trials. While there were no clear differences in the reasons for MPA dose reduction, interruption, or discontinuation between the EC-MPS and MMF cohorts, the observation that there were no marked differences between the groups in the incidence of any category of adverse event (including gastrointestinal events) despite the significantly higher doses of MPA in the EC-MPS group suggests that MMF dose may have been reduced to a greater extent to achieve a similar level of tolerance. It should be borne in mind, however, that standard adverse event reporting of the type used in this study may not be sufficiently sensitive to detect differences in the gastrointestinal symptom burden between the two MPA formulations. Randomized trials of EC-MPS vs. MMF in *de novo* 30 and maintenance 31 kidney transplant recipients that used standard adverse event reporting techniques also observed no marked differences in gastrointestinal adverse event rates between the two formulations, while patient-reported outcome data have suggested a lower gastrointestinal symptom burden with EC-MPS 32.

This large-scale analysis offers the advantage of prospective data collection in real-life clinical practice, where immunosuppressive dosing is not protocol-driven and the study population was not selected other than the specification that patients were receiving tacrolimus and MPA. Unfortunately, the small number of patients receiving a baseline MPA dose >2000 mg (n = 23) prohibited a meaningful consideration of this regimen. We are also aware that MPA blood concentration was not recorded and that no data were collected regarding the reasons for selection of baseline MPA dose. While every attempt was made statistically to correct for potential bias, residual bias may have remained. Lastly, as an observational study, patients and physicians were aware of whether EC-MPS or MMF was being administered, representing a potential source of bias.

In this, the first prospective analysis to consider the impact of baseline MPA dose in tacrolimus-treated kidney transplant patients, an initial dose of <2000 mg was not associated with inferior immunosuppressive efficacy although neither was a safety benefit observed in this cohort. Dose reductions from the recommended starting dose of 2000 mg were made in approximately 40% of patients, but patients receiving EC-MPS were significantly more likely to remain on 2000 mg and tolerated a higher mean MPA dose without an increase in adverse events. In conclusion, this non-interventional study suggests that an initial MPA dose below the recommended dose of 2000 mg may be effective in *de novo* kidney transplant patients receiving standard tacrolimus-based immunosuppression, but controlled dose-finding studies are necessary to confirm this result and to identify a lower threshold for initial MPA dosing.

## References

[b1] European Mycophenolate Mofetil Study Group (1995). Placebo-controlled study of mycophenolate mofetil combined with cyclosporin and corticosteroids for prevention of acute rejection. Lancet.

[b2] Sollinger HW, US Renal Transplant Mycophenolate Mofetil Study Group (1995). Mycophenolate mofetil for the prevention of acute rejection in primary cadaveric renal allograft recipients. Transplantation.

[b3] The Tricontinental Mycophenolate Mofetil Renal Transplantation Study Group (1996). A blinded, randomized clinical trial of mycophenolate mofetil for the prevention of acute rejection in cadaveric renal transplantation. Transplantation.

[b4] Cellcept® Prescribing Information

[b5] Meier-Kriesche HU, Li S, Gruessner RW (2006). Immunosuppression: evolution in practice and trends, 1994–2004. Am J Transplant.

[b6] Van Gelder T, Shaw LM (2005). The rationale for and limitations of therapeutic drug monitoring for mycophenolate mofetil in transplantation. Transplantation.

[b7] Kaplan B, Meier-Kriesche HU, Minnick P (2005). Randomized calcineurin inhibitor cross over study to measure the pharmacokinetics of co-administered enteric-coated mycophenolate sodium. Clin Transplant.

[b8] Park JM, Lake KD, Cibrik DM (2008). Impact of changing from cyclosporine to tacrolimus on pharmacokinetics of mycophenolic acid in renal transplant recipients with diabetes. Ther Drug Monit.

[b9] Grinyó JM, Ekberg H, Mamelok RD (2009). The pharmacokinetics of mycophenolate mofetil in renal transplant recipients receiving standard-dose or low-dose cyclosporine, low-dose tacrolimus or low-dose sirolimus: the Symphony Pharmacokinetic Substudy. Nephrol Dial Transplant.

[b10] Gourishankar S, Houde I, Keown P (2010). The CLEAR Study: a 5-day, 3-g loading dose of mycophenolate mofetil versus standard 2-g dosing in renal transplantation. Clin J Am Soc Nephrol.

[b11] Knoll GA, Macdonald I, Khan A, Van Walraven C (2003). Mycophenolate mofetil dose reduction and the risk of acute rejection after renal transplantation. J Am Soc Nephrol.

[b12] Tierce JC, Porterfield-Baxa J, Petrilla AA, Kilburg A, Ferguson RM (2005). Impact of mycophenolate mofetil (MMF)-related gastrointestinal complications and MMF dose alterations on transplant outcomes and healthcare costs in renal recipients. Clin Transplant.

[b13] Shah S, Collett D, Johnson R, Raftery M, Rudge C, Yaqoob MM (2008). The effect of mycophenolate mofetil and azathioprine dose on renal allograft outcome in the United Kingdom. Transplantation.

[b14] Hardinger KL, Brennan DC, Lowell J, Schnitzler MA (2004). Long-term outcome of gastrointestinal complications in renal transplant patients treated with mycophenolate mofetil. Transplant Int.

[b15] Machnicki G, Ricci JF, Brennan DC, Schnitzler MA (2008). Economic impact and long-term graft outcomes of mycophenolate mofetil dosage modifications following gastrointestinal complications in renal transplant recipients. Pharmacoeconomics.

[b16] Bunnapradist S, Lentine KL, Burroughs TE (2006). Mycophenolate mofetil dose reductions and discontinuations after gastrointestinal complications are associated with renal transplant graft failure. Transplantation.

[b17] Opelz G, Döhler B (2008). Effect on kidney graft survival of reducing or discontinuing maintenance immunosuppression after the first year posttransplant. Transplantation.

[b18] Shehata M, Bhandari S, Venkat-Raman G (2009). Effect of conversion from mycophenolate mofetil to enteric-coated mycophenolate sodium on maximum tolerated dose and gastrointestinal symptoms following kidney transplantation. Transpl Int.

[b19] Arns W, Breuer S, Choudhury S (2005). Enteric-coated mycophenolate sodium delivers bioequivalent MPA exposure compared with mycophenolate mofetil. Clin Transplant.

[b20] Cox DR (1972). Regression models and life-tables (with discussion). J R Stat Soc Series B Meth.

[b21] Hosmer DW, Lemeshow S (1999). Applied Survival Analysis: Regression Modeling of Time to Event Data.

[b22] Fitzmaurice GM, Laird NM, Ware JH (2004). Applied Longitudinal Analysis.

[b23] Poggio ED, Wang X, Weinstein DM (2006). Assessing glomerular filtration rate by estimation equations in kidney transplant recipients. Am J Transplant.

[b24] Zucker K, Tsaroucha A, Olson L, Esquenazi V, Tzakis A, Miller J (1999). Evidence that tacrolimus augments the bioavailability of mycophenolate mofetil through the inhibition of mycophenolic acid glucuronidation. Ther Drug Monit.

[b25] Vincenti F, Friman S, Scheuermann E (2007). Results of an international, randomized trial comparing glucose metabolism disorders and outcome with cyclosporine versus tacrolimus. Am J Transplant.

[b26] van Gelder T, Hilbrands LB, Vanrenterghem Y (1999). A randomized double-blind, multicenter plasma concentration controlled study of the safety and efficacy of oral mycophenolate mofetil for the prevention of acute rejection after kidney Transplantation. Transplantation.

[b27] Arns W (2007). Non-infectious gastrointestinal (GI) complications of mycophenolic acid therapy: a consequence of local GI toxicity?. Transplant Proc.

[b28] Bilodeau JF, Montambault P, Wolff JL, Lemire J, Masse M (2009). Evaluation of tolerability and ability to increase immunosuppression in renal transplant patients converted from mycophenolate mofetil to enteric-coated mycophenolate sodium. Transplant Proc.

[b29] Massari P, Duro-Garcia V, Girón F, MyPROMS LatAm Study Group (2005). Safety assessment of the conversion from mycophenolate mofetil to enteric-coated mycophenolate sodium in stable renal transplant recipients. Transplant Proc.

[b30] Salvadori M, Holzer H, de Mattos A, ERL B301 Study Groups (2004). Enteric-coated mycophenolate sodium is therapeutically equivalent to mycophenolate mofetil in de novo renal transplant patients. Am J Transplant.

[b31] Budde K, Curtis J, Knoll G, ERL B302 Study Group (2004). Enteric-coated mycophenolate sodium can be safely administered in maintenance renal transplant patients: results of a 1-year study. Am J Transplant.

[b32] Chan L, Mulgaonkar S, Walker R (2006). Patient-reported gastrointestinal symptom burden and health-related quality of life following conversion from mycophenolate mofetil to enteric-coated mycophenolate sodium. Transplantation.

